# Exploring burnout: An online survey among diverse nursing teams in Germany

**DOI:** 10.1016/j.ijnsa.2026.100661

**Published:** 2026-08-13

**Authors:** Anna Maria Khouri, Jan C. Zoellick, Lisa Peppler, Liane Schenk, Anna Schneider

**Affiliations:** Charité – University Medical Center Berlin, corporate member of Freie Universität Berlin and Humboldt-Universität zu Berlin, Institute of Medical Sociology and Rehabilitation Science, Charitéplatz 1, 10117, Berlin, Germany

**Keywords:** Burnout, Hospital, Nursing staff, Operations research, Personnel staffing and scheduling

## Abstract

**Background:**

Immigration and the increasing shift toward nursing as a university degree lead to greater diversity in German nursing teams and have an impact on cooperation in everyday nursing care. Studies on the interactions between psychosocial working conditions and individual diversity characteristics on the mental health of nurses are scarce.

**Objective:**

This study examines the relationships between psychosocial working conditions of nurses (psychological safety in the team, cooperation with physicians, leadership quality, staffing on the ward) and individual sociodemographic and occupational characteristics (age, gender, migration experience, qualifications, management responsibility, number of close personal contacts, partnership, parental status) of nurses with burnout.

**Design:**

The data basis is a cross-sectional online survey which was conducted as part of a mixed-methods study. Validated questionnaire instruments (e.g. COPSOQ, PES-NWI-R, PS-C) were used to assess the general work situation of nursing staff and to measure personal burnout.

**Setting:**

Inpatient care teams in two healthcare companies with several hospitals in a major German city.

**Participants:**

684 nurses of legal age with high diversity in terms of countries of origin, (formal) qualification profiles and other social differentiations such as age, gender, and ethnicity.

**Methods:**

In addition to descriptive and bivariate analyses, linear blockwise regressions were performed to identify stepwise associations of working conditions and sociodemographic variables with burnout. Statistical analyses were performed using IBM SPSS Statistics (version 29.0).

**Results:**

N = 632 nurses were included in the analysis. The participants were predominantly female (76%), on average 44 years old, born in Germany (88%) and had three or more years of professional training (78%). Factors that were associated with lower burnout rates were a more positive assessment of psychological safety in their own nursing team (β = -0.16, p = .003) and a more positive assessment of staffing levels with nurses on their own ward (β = -0.32, p ≤ .001). Having one to two close relations in the personal environment had a negative impact on one`s personal burnout (β = 0.12, p = .046) in comparison to six or more close contacts. The other characteristics examined were not statistically significant in multivariable regression analyses.

**Conclusions:**

Social support in the immediate care team and sufficient staffing were associated with the mental health of nurses. In day-to-day nursing care, improvements in cooperation within the core team and an adequate staffing ratio appear to enable a reduction in occupational stressors for nursing staff. Interactions of the factors investigated with other occupational and private stressors require future research.

**Tweetable abstract:**

Better collaboration and proper staffing ratios in diverse nursing teams help reduce burnout in everyday nursing practice.

**What is already known**

• Diversity in nursing teams in Germany is rising due to staff shortages, nurse migration, and the increasing academic requirements of the profession.
• Strong intraprofessional teamwork is linked to improved patient care.
• There is limited understanding of how a nurse’s own diverse background influences their perceptions and behaviours in team settings.

Alt-text: Unlabelled box dummy alt text

**What this paper adds**

• Enhancements in collaboration within the nursing team and an appropriate staffing ratio are associated with a reduction of burnout for nursing personnel in routine nursing practice.
• The diversity dimensions we have examined in our study were not associated with burnout rates among the participating nurses.

Alt-text: Unlabelled box dummy alt text

## 1 Background

The evolving landscape of nursing care in Germany is increasingly shaped by immigration (Theobald, 2022) and the partial shift from nursing as a three-year training program toward a university degree (Kälble, 2013), resulting in a notable diversification of care teams.

In 2024, the number of foreign employees in Germany working in the care sector exceeded 300,000 making up 18 percent of the total nursing staff of the 1.7 million total nursing personnel (Federal Employment Agency, 2025). According to this information from the Federal Employment Agency, the growth in the number of people working in care in Germany since 2022 is exclusively due to foreign personnel (Federal Employment Agency, 2025).

This transformation is anticipated to have profound implications for intra- and interprofessional collaboration within daily nursing practices (Stagge, 2020). As healthcare environments become more heterogeneous, understanding the dynamics of these diverse care teams is essential for fostering effective collaboration and enhancing the overall quality of patient care.

The working atmosphere, social support in the workplace, staffing levels and inter- and intradisciplinary collaboration within the nursing team and with physicians influence the mental health and job satisfaction of nursing staff (Högstedt et al., 2024). Previous studies have reported differences in social interaction between migrant and non-migrant nurses (Schmidt and Uman, 2024) as well as stereotyping in the workplace with regard to age and gender (Teresa-Morales et al., 2022). Migrant nurses often face unique challenges as they adapt to new working environments, professional cultures, and organizational structures in their host countries (Pütz et al., 2019).

The trend towards greater academic qualifications for nurses has been another defining aspect of the profession's evolution. Currently, nursing training in Germany is a profession with a three-year training program that is defined by legal regulations and is now slowly undergoing a shift toward higher education (Kälble, 2013).

Additionally, studies have shown that attitudes toward the academic training of nurses vary widely, with nurses holding university degrees or nursing students expressing a more positive view of the increasing professionalization of the nursing profession (Mertens et al., 2019; Ehrenbrandtner, 2022).

The dynamics of migration, the professionalization of nursing, and the challenges of burnout intersect in complex and consequential ways within the German healthcare system which will be described in the following parts.

## 2 Burnout and well-being: consequences for nurses

Job burnout is a psychological syndrome resulting from prolonged exposure to work-related stress, characterized by three core dimensions: emotional exhaustion (feeling drained by work), depersonalization (developing a cynical, detached attitude towards work and others), and reduced personal accomplishment (a sense of ineffectiveness and reduced competence) (Maslach et al., 2001).

Mental health and especially the rising incidence of burnout among nurses are particularly important topics and becoming increasingly important in the healthcare sector (Sullivan et al., 2022). Burnout relates to a decrease in the job satisfaction of the individual nursing staff (Quesada-Puga et al., 2024) and cooperation in the respective nursing teams (Zhao et al., 2025).

The rising incidence of burnout among nursing staff poses a significant threat not only to individual practitioners but also to patient outcomes and overall healthcare efficacy (Li et al., 2024).

The consequences of burnout in nursing extend beyond individual well-being to affect the overall healthcare system. Increased burnout is associated with reduced job satisfaction, lower productivity, and a higher likelihood of premature career endings (Jun et al., 2021). Research has shown that unsatisfactory working conditions - such as lack of family compatibility, high work intensity, and lack of recognition from superiors - are key drivers of burnout and turnover in the nursing workforce (Maglalang et al., 2021; Alahiane et al., 2023).

## 3 Work organization and interprofessional collaboration

The organization of work and the dynamics of interprofessional collaboration are central to understanding the burnout experience in nursing. Research has consistently shown that high levels of job strain, lack of support, and poor work organization are significant predictors of burnout among nurses (Getie et al., 2025). Specifically, poor working conditions, such as understaffing, irregular hours, and limited control over work schedules combined with a strong work ethic, are major contributors to burnout (Dixon-Woods et al., 2024).

Moreover, interprofessional collaboration - particularly between nurses, physicians and other healthcare professionals - plays a crucial role in reducing work-related stress and improving job satisfaction (Kaiser et al., 2018). Collaborative and supportive work environments are associated with better mental health outcomes for nurses, including lower rates of burnout (Chiminelli-Tomás et al., 2025). Inadequate support from supervisors, as well as perceived low recognition of one’s work, may further undermine professional satisfaction and contribute to internal resignation or the decision to leave the profession entirely (Hämmig, 2017).

## 4 Migration and the professionalization of nursing

According to the state of the world´s nursing report from the World Health Organization (WHO) in 2020, particularly high-income countries rely on migrant nurses as a pillar of healthcare provision with rates of 15% of all nurses being foreign-born or foreign-trained (WHO, 2020). However, migrant nurses often experience a range of challenges in their new environments, such as cultural and linguistic barriers, difficulties in recognizing foreign qualifications, and adjusting to new healthcare standards and practices (Kamau et al., 2022; Choi et al., 2019). These stressors can increase the likelihood of burnout, as migrant nurses are often tasked with not only providing care but also navigating the complexities of a foreign healthcare system (Atta et al., 2025). Moreover, hierarchical and fragmented work environments, which are often exacerbated by the integration of migrant nurses into pre-existing teams, can hinder collaboration and increase stress levels (Kiptulon et al., 2024)

In many countries, nursing education has increasingly moved toward a university-based model, emphasizing advanced clinical skills, leadership training, and evidence-based practice (Al-Worafi, 2024). While this shift aims to enhance the professionalism and autonomy of nurses (Pursio et al., 2021), it has also led to increased job expectations, which can contribute to work-related stress and burnout (Bunjak et al., 2023).

## 5 Aim of the study

Despite these insights, gaps remain in our understanding of how specific diversity factors - such as age, gender, migration history, and the level of academic training - interact to influence these outcomes. Although previous research has established that psychosocial work conditions are important determinants of nurses' well-being and burnout, and that individual and diversity-related characteristics may also be associated with burnout, these bodies of literature have largely developed in parallel. Few studies combine a number of diversity-related factors and the psychosocial work environment together in relation to burnout among hospital nurses. As a result, it is not yet clear if diversity-related characteristics are linked to burnout independently of psychosocial work environments and if particular groups of nurses may be more at risk of adverse psychosocial working conditions. It is important to address these questions, as approaches that disregards workforce diversity may not fully reflect the unique experiences of nurses in the workplace.

Studies in Germany are largely missing, and international studies are only partially indicative, because nursing is state-regulated and thus context-dependent.

This study seeks to bridge existing research gaps by exploring the associations between the identified diversity factors and their impacts on intra- and interprofessional collaboration and burnout within nursing teams in Germany. By elucidating these relationships, we aim to provide insights that can inform policy and practice, ultimately contributing to improved working conditions and patient care quality in Germany's evolving healthcare context.

## 6 Methods

### 6.1 Data collection and participants

“Care in Transition - Care teams in the field of tension between migration and academization using the example of hospitals and nursing homes” (CareTrans) was a prospective, non-interventional, multicenter mixed-methods study to investigate communication and team structures in diverse care teams. This paper is based on the quantitative part of the study, which consisted of an online survey of nurses in two large hospital chains in a major German city.

Prior to the online survey, we conducted qualitative and quantitative pretests to review the survey with professionals from the field. As part of the quantitative pre-test, we asked two nursing teams from one participating hospital to complete the entire questionnaire in an initial online version. Between May and August 2023, we invited all nursing staff in 11 hospitals and 17 nursing homes to participate in the online survey. The invitation was forwarded by nursing supervisors to the hospitals’ distribution lists by email via link to the work address of all nurses and was also published on the intranet of both hospital chains. To increase the response rate, we sent two reminders and printed out flyers that were distributed across hospitals and nursing homes.

The survey was administered using the software Unipark. The data collected was stored on an external server, the protection of which is guaranteed by the Unipark provider QuestBack. We enabled participation in German and English. The survey was translated by a professional interpreter.

The study population consisted of approximately 11,400 nursing staff members who, according to publicly available statistics from the participating hospital operators, were employed there at the time of data collection.

No additional demographic information was available on the total population of eligible nurses for non-responder analysis.

### 6.2 Measures and variables of the survey

The survey contained 37 items divided into the following subject areas: professional biography, sociodemographics, burnout and wellbeing, leadership and teamwork, personnel equipment, and psychological safety. We primarily used validated scales used previously in studies with nurses as target groups.

#### 6.2.1 Professional biography

We collected information about the professional background of participating nurses, especially regarding the qualification (1-2 years vocational training / 3 years vocational training / university degree) as well as years of work experience and leadership position (yes/no).

#### 6.2.2 Sociodemographics

When selecting questions to measure sociodemographic variables, we used formulations from German-language surveys representative of the population, which record the relevant information in a diversity-sensitive manner, such as the Current State of Health in Germany study (GEDA 2019/2020-EHIS) and the survey of the National Discrimination and Racism Monitor (NaDiRa). We reviewed all formulations of questions that did not originate from validated instruments and adapted where necessary to strengthen their relevance in the nursing context. Next to the diversity factors age, gender and migration experience, we obtained information on relationship status (yes/no), children (yes/no), being a single parent (yes/no), and social embedding measured via the number of people so close to oneself that one can rely on them.

#### 6.2.3 Burnout and wellbeing

Burnout as our primary outcome refers to a state of prolonged physical, emotional, and mental exhaustion resulting from chronic work stress according to the Copenhagen Burnout Inventory (Kristensen et al., 2005). We used the Copenhagen Burnout Inventory (CBI) which is integrated in the Copenhagen Psychosocial Questionnaire COPSOQ (Nübling et al., 2005) to measure burnout with three items on five-point Likert scales ranging between never (1) and always (5). An example item was “How often do you feel emotionally exhausted?”. This scale and all other scales used from the COPSOQ were converted to a scale of 0–100. A high score indicated high levels of burnout. In the present study, the scale demonstrated good internal consistency (Cronbach’s ∝= .85).

Further questions assessed the occurrence of presentism, work satisfaction and general state of health of the participants.

#### 6.2.4 Assessment of leadership

The assessment of leadership was measured using the COPSOQ with four items on five-point Likert scales ranging between to a very large extent (1) to a very small extent (5). An example item was “To what extent would you say that your immediate superior is good at work planning?”. We recoded the items so that higher scores indicated a positive assessment of leadership. Analysis of the scale in the current sample indicated high internal consistency (Cronbach’s ∝= .92).

#### 6.2.5 Interprofessional teamwork

Interprofessional teamwork was assessed using five items from the Practice Environment Scale of the nurse work environment index (revised) (PES-NWI-R) (Swiger et al., 2017), rated on a four-point Likert scale ranging between agree (1) to disagree (4). These items measured the level of collaboration and mutual respect between the two professional groups. An example item was **“**Physicians and nursing staff work closely together as a team.” We recoded the items so that higher scores indicated positive assessments of teamwork quality, i.e. cooperative and respectful interactions. The internal consistency of the scale was good (Cronbach’s ∝= .92).

#### 6.2.6 Personnel equipment

Personnel equipment was assessed using four items from the Practice Environment Scale of the nurse work environment index (revised) (PES-NWI-R), rated on a four-point Likert scale ranging between agree (1) to disagree (4). These items measured the nurses’ perceptions about whether their ward had sufficient personnel and resources to meet the needs of patients and deliver quality care, as these factors directly influence their workload and capacity for effective teamwork. An example item was “There are adequate support services that allow me to spend time with my patients.” We recoded the items so that a higher score indicated a more positive assessment of personnel equipment. The scale proved to be highly reliable in the present cohort (Cronbach’s ∝= .83).

#### 6.2.7 Psychological safety

The psychological safety in a team describes the shared perception of team members that the team is safe for interpersonal risk-taking and that speaking up will not result in negative consequences (Fischer and Hüttermann, 2020). Using the PsySafety-Check (PS-C), we measured this construct with seven items on a five-point Likert scale ranging between disagree (1) to agree (5). An example item was “Members of this nursing team are able to bring up problems and tough issues.” We recoded the items so that a higher score indicated a more positive assessment of psychological safety. The scale showed reliable internal consistency (Cronbach’s ∝= .82).

### 6.3 Data analysis

To answer our research question, we used descriptive statistics, χ²-tests for nominal variables, t-tests and ANOVAs for group comparisons, and regression analyses for directed relationships. We recoded all scales for the standardization of the calculation so that the lowest value corresponds to 1 and the highest value to 4, 5 or 100 depending on the scale. We modelled a hierarchical regression model for the outcome burnout. In the first step, we included individual diversity characteristics of the nursing staff, i.e. gender (male/female), age (continuous), country of birth (Germany/other), and qualification level (1-2 years vocational training/3 or more years vocational training/university degree). This initial step aimed to control for demographic and professional background differences among the nursing personnel.

In the second step, psychosocial working conditions were added to the model. These included the continuous variables leadership quality, psychological safety, interprofessional teamwork, and the availability of personnel equipment, as well as leadership role (yes/no). This allowed for the assessment of how workplace environment and social factors contributed to the outcome beyond individual characteristics.

In the third and final step, private life circumstances of the nursing staff were included. Variables in this block were the number of close social contacts (None/1-2/3-5/6 or more), partnership status (yes/no), and parenthood (yes/no). By adding these factors, the analysis accounted for how personal life situations might further explain variations in the outcome.

This hierarchical approach provided a comprehensive framework to evaluate the relative impact of individual characteristics, work environment factors, and private life conditions on the dependent variable within the regression model.

All statistical analyses followed a pre-specified plan and were conducted using IBM SPSS Statistics 29.0. Unless otherwise specified, we set the significance level for all analyses to α =.05.

The dataset contained 16.3% missing data on the relevant independent and dependent variables. We calculated Little’s MCAR on the target population of N = 684, which was not significant (χ²= 1949,18, p= 0.11), indicating that the missing data were missing completely at random (MCAR). Therefore, complete case analysis was applied in the subsequent analyses, minimizing bias due to missingness.

### 6.4 Ethical approval and informed consent

The CareTrans study received a positive ethics vote from the Ethics Committee of Charité - University Medical Center Berlin (EA1/260/21) on 22.10.2021. The data protection concept was positively reviewed by the data protection officer of Charité - University Medical Center Berlin.

Participation in the study was voluntary and was based on extensive written information provided in advance. The online survey was conducted anonymously.

## 7 Results

### 7.1 Study population

*N* = *867* answered at least one item before dropping out. After adjusting the number of participants to only those who met the inclusion criteria, which are being eligible to work in nursing care and being of legal age as well as answering more than one question in the survey, the total sample of the survey included *N* = *684.* We then adjusted our sample and did not include those participants in the following analyses who did not answer the minimum number of items required to obtain meaningful results. These so-called dropouts have not fulfilled the minimum requirements to answer the first scale in the questionnaire. This left us with a final sample of *N* = *632.* The final response rate is therefore around 6%.

Table 1 shows descriptive statistics of respondents’ characteristics, further stratified into gender categories. We omitted diverse gender from further analyses due to the small sample size (n = 7).

**Table 1:** Characteristics of participants.

	Total	Male	Female	Diverse
N	632 (100%)	126 (23%)	413 (76%)	7 (1%)
Age (M, SD)	44.2 (11.6)	42.3 (10.6)	44.7 (11.9)	40.9 (13.3)
Country of birth				
Germany	487 (88%)	105 (83%)	369 (90%)	6 (86%)
Other	65 (12%)	21 (17%)	43 (10%)	1 (14%)
Qualification				
Vocational training (1-2 years)	24 (4%)	7 (6%)	15 (4%)	0
Vocational training (3 or more years)	418 (78%)	87 (72%)	319 (79%)	6 (86%)
University or College degree	98 (18%)	27 (22%)	68 (17%)	1 (14%)
Years of work experience (M, SD)	20.2 (12.8)	18.1 (11.1)	21.9 (13.0)	14.7 (12.6)
Leadership position				
Yes	129 (23%)	31 (25%)	97 (24%)	0
No	426 (77%)	95 (75%)	315 (76%)	7 (100%)
Children				
Yes	319 (59%)	60 (48%)	251 (62%)	2 (33%)
No	226 (41%)	65 (52%)	156 (38%)	4 (77%)
Relationship status				
Yes	397 (73%)	88 (70%)	301 (75%)	3 (50%)
No	144 (27%)	37 (30%)	102 (25%)	3 (50%)
Close relationships				
None	17 (3%)	8 (7%)	9 (3%)	0
One to two	148 (31%)	33 (30%)	109 (30%)	2 (40%)
Three to five	219 (46%)	51 (47%)	163 (46)	2 (40%)
Six or more	95 (20%)	18 (16%)	76 (21%)	1 (20%)

Notes: N= 632; M Mean. SD Standard deviation

Bivariate analyses as seen in Table 2, Table 3 revealed multiple associations between individual and organizational characteristics and psychological safety in the team, assessment of leadership as well as burnout and interprofessional teamwork.

**Table 2:** Differences in the scales regarding the diversity dimensions.

	N	Burnout (M, SD)	Leadership assessment (M, SD)	Interprofessional teamwork (M, SD)	Personnel equipment (M, SD)	Psychological safety (M, SD)
Gender^a^						
Male	126	59.8 (18.7)	57.1 (23.9)	2.82 (0.68)	2.23 (0.69)	3.50 (0.78)
Female	413	56.3 (21.5)	63.0 (25.3)	2.92 (0.75)	2.33 (0.75)	3.58 (0.84)
County of birth^a^						
Germany	487	58.6 (19.6)	59.1 (24.2)	2.84 (0.69)	2.26 (0.71)	3.56 (0.79)
Other	65	62.6 (19.3)	53.9 (24.9)	2.87 (0.73)	2.18 (0.69)	3.15^⁎⁎⁎^ (0.75)
Qualification^b^						
Vocational training (1-2 years)	24	55.9 (22.6)	59.9 (27.3)	2.65 (0.69)	2.32 (0.67)	3.41 (0.83)
Vocational training (3 or more years)	418	59.4 (19.2)	57.9 (23.9)	2.86 (0.69)	2.22 (0.71)	3.54 (0.80)
University or College degree	98	59.9 (21.25)	58.5 (25.6)	2.82 (0.73)	2.32 (0.73)	3.40 (0.80)
Relationship status^a^						
Yes	397	58.5 (19.1)	58.1 (24.6)	2.84 (0.68)	2.25 (0.70)	3.52 (0.77)
No	144	60.6 (20.7)	59.7 (23.4)	2.90 (0.75)	2.27 (0.72)	3.52 (0.87)
Children^a^						
Yes	319	57.8 (18.6)	60.0 (24.2)	2.89 (0.66)	2.28 (0.70)	3.57 (0.76)
No	226	60.7 (20.8)	56.6 (24.3)	2.80 (0.74)	2.22 (0.72)	3.44 (0.83)
Close relationships^b^						
None	17	66.4 (26.1)	54.0 (25.6)	2.80 (0.88)	2.16 (0.83)	2.92 (0.76)
One to two	147	62.1 (19.5)	54.3 (25.0)	2.67 (0.75)	2.16 (0.65)	3.33 (0.75)
Three to five	219	58.9 (17.9)	59.0 (23.3)	2.94^⁎⁎^ (0.69)	2.24 (0.73)	3.53 (0.78)
Six or more	95	53.2^⁎⁎^ (20.1)	67.0^⁎⁎^ (23.4)	2.92 (0.66)	2.38 (0.63)	3.91^⁎⁎⁎^ (0.70)

Notes: Statistically significant association parameters are printed in bold *N* = 632; *M* mean. *SD* standard deviation. ^a^independent *t* test; ^b^one-way ANOVA with post-hoc Bonferroni tests. *p < .05, **p < .01, ***p ≤ .001

**Table 3:** Correlations between the primary outcome burnout and organizational as well as sociodemographic variables.

		1	2	3	4	5	6
1	Age	-					
2	Burnout	-.08	-				
3	Leadership assessment	.02	-.25^⁎⁎⁎^	-			
4	Interprofessional teamwork	.00	-.22^⁎⁎⁎^	.21^⁎⁎⁎^	-		
5	Personnel equipment	.04	-.40^⁎⁎⁎^	.33^⁎⁎⁎^	.31^⁎⁎⁎^	-	
6	Psychological safety	.09*	-.32^⁎⁎⁎^	.56^⁎⁎⁎^	.27^⁎⁎⁎^	0.29^⁎⁎⁎^	-

Notes: Pearson’s correlation coefficient. *N* = 632; *p < .05, **p < .01, ***p ≤ .001

The migration background of the nurses as well as the number of close relationships were associated with the assessment of psychological safety in the team. Therefore, being born in Germany and having six or more close relationships tended to be associated with a higher rating for psychological safety. Having six or more close relationships was also associated with burnout and the assessment of leadership. Participants who had three to five close relationships to rely on were associated with higher rates of interprofessional teamwork. All other variables did not show associations.

Most correlations among the metric study variables were significant.

### 7.2 Regression analyses

The overall directed model was significant, *F* (15, 423)= 8.81, *p* < .001, and explained approximately 24% of the variance in burnout (R²= .238). The first block in Table 4 focused on diversity dimensions, revealing a significant association between gender and burnout, with significant associations with the male gender (β = -0.10; *p* = .035). Age, country of birth, and qualification did not show statistical significance. The second block further examined work organization variables, highlighting that psychological safety in the team (β = -0,19; *p* *<* *.*001) and personnel equipment (β = -0,32; *p* *<* .001) influenced burnout positively. Interprofessional teamwork, leadership quality, and one's own position had no statistical significance. Furthermore, the influence of the variable gender lost significance in the second block. The third block incorporated private and social variables, expanding the analysis further. The results underscore the importance of psychological safety (β = -0,16; *p* = .003) and sufficient personnel equipment (β = -0,32; *p* *<* .001) in lower burnout levels among professionals, while other factors like interprofessional teamwork and leadership quality did not show significance. Regarding the social factors, close relations with one to two people in the personal environment seem to have a more negative impact on one`s personal burnout (β = 0,12; *p* = .046) in comparison to the reference category of six or more close relations in this directed analysis.

**Table 4:** Multiple blockwise linear regression analyses of individual and organizational variables (independent variables) on personal burnout (dependent variables).

	Block 1				Block 2				Block 3			
	*B*	SE	*ß*	95% CI (for *B)*	*B*	SE	*ß*	95% CI (for *B*)	B	SE	*ß*	95% CI (for *B*)
Constant	61.52	5.19		51.3; 71.7	109.53	7.63		94.5; 124.5	101.33	8.81		84.0; 118.7
Gender (female)	ref				ref				ref			
Gender (male)	-4.60	2.17	-0.10*	-8.9; -0.3	-2.27	1.97	-0.05	-6.1; 1.6	-2.79	2.00	-0.06	-6.7; 1.1
Age	-0.11	0.09	-0.07	-0.3; 0.1	-0.10	0.08	-0.06	-0.2;0.1	-0.07	0.08	-0.04	-0.2; 0.1
Country of birth (Germany)	ref				ref				ref			
Country of birth (other)	2.28	1.58	0.07	-0.8; 5.4	0.84	1.45	0.03	-2.0; 3.7	0.66	1.47	0.02	-2.2; 3.5
Qualification (University or college degree)	ref				ref				ref			
Qualification (Vocational training (1-2 years)	-3.42	4.99	-0.04	-13.2; 6.4	-2.33	4.57	-0.02	-11.3; 6.7	-2.29	4.59	-0.02	-11.3; 6.7
Qualification (Vocational training (3 or more years)	1.05	2.57	0.02	-4.0; 6.1	0.65	2.40	0.01	-4.1; 5.4	0.80	2.41	0.02	-3.9; 5.5
Leadership assessment^1^					-0.05	1.03	0.00	-2.1; 2.0	-0.03	1.03	0.00	-2.1; 2.0
Leadership position (yes)					ref				ref			
Leadership position (no)					-1.92	2.11	-0.04	-6.1; 2.2	-2.57	2.14	-0.06	-6.8; 1.6
Psychological safety^3^					-4.61	1.32	-0.19^⁎⁎⁎^	-7.2; -2.0	-4.02	1.35	-0.16^⁎⁎^	-6.7; -1.4
Interprofessional teamwork^2^					-2.42	1.27	-0.09	-4.9; 0.1	-2.37	1.29	-0.09	-4.9; 0.2
Personnel equipment^2^					-9.19	1.36	-0.32^⁎⁎⁎^	-11.9; -6.5	-9.19	1.36	-0.32^⁎⁎⁎^	-11.9; 6.5
Close relations (6 or more)									ref			
Close relations (none)									6.19	5.14	0.06	-3.9; 16.3
Close relations (1 to 2)									4.95	2.48	0.12*	0.1; 9.8
Close relations (3 to 5)									3.87	2.23	0.10	-0.5; 8.2
Relationship (yes)									ref			
Relationship (no)									1.32	2.02	0.03	-2.6; 5.3
Parent (yes)									ref			
Parent (no)									0.86	1.91	0.02	-2.9; 4.6

Notes: Statistically significant association parameters are printed in bold. *N* = 632; *B* unstandardized coefficient, *SE* standard error*, β* standardized coefficient, *CI* confidence interval; ^1^*COPSOQ* Copenhagen Psychosocial Questionnaire, ^2^*PES-NWI-R* Practice Environment Scale of the Nursing Work Index revised, ^3^*PS-C* PsySafety-Check; *p < .05, ** *p* < .01, ***p ≤ .001

## 8 Discussion

Our study investigated the associations of nurse’s diversity factors, intra- and interprofessional collaboration, personnel equipment and social factors with burnout among nursing staff using survey results from 632 participants among hospitals and nursing homes in a major city in Germany.

The results of our study indicated that working conditions on the ward were associated with the likelihood of burnout among nurses.

We found out that the higher psychological safety within the team and better personnel equipment at the ward as well as having a higher number of close personal relationships were associated with lower burnout rates among nurses.

These findings highlight the critical importance of collaborative practice environments in mitigating occupational stressors inherent to healthcare settings. Interestingly, the diversity dimensions we have examined in our study were not associated with burnout rates among the participating nurses.

### 8.1 Possible interpretations and implications

Our main results indicated that factors associated with lower burnout rates among the participating nurses were a more positive assessment of psychological safety in their own nursing team and a more positive assessment of personnel equipment with nurses on their own ward. Furthermore, having a broader number of close relationships who oneself can rely on was also associated with lower burnout rates. A considerable number of studies conducted after the COVID-19 pandemic found similar results in differing countries, where due to staffing shortages and long working hours with limited medical supplies and resources rising rates of burnout were reported (Nantsupawat et al., 2024; Abdulmohdi, 2024). Abdulmohdi (2024) conducted a cross-sectional survey in the UK showing that stronger social support during the pandemic helped nurses become more resilient, with resilience functioning as a mediator in the relationships between perceived social support and burnout. According to them, “social support provides a sense of belonging, validation and reassurance that helps nurses maintain a positive outlook, feel less isolated and cope better with stress and emotional exhaustion” (p.8).

Wood et al. (2022) reported similar results, drawing a clear connection between social support and burnout, highlighting the negative effects of loneliness on nurses. The effects of COVID-19 could also play a role in our results due to adverse effects of working during the pandemic on nurses’ physical (e.g., fatigue, sleeplessness) and psychosocial (e.g., post-traumatic stress disorder, depression) well-being (Chemali et al., 2022). To address the long-term impact of COVID-19 on nurse burnout, post-pandemic healthcare organizations will need to implement sustained mental health support, workload adjustments, and improved staffing.

In the diversity dimensions we examined, only gender played a role in psychological stress, while the other dimensions in our study did not show any statistically significant associations, thus taking on a more minor role. This effect was not stable in our results and should therefore be carefully interpreted. Women tended to show higher rates of burnout in comparison to men. Although this relationship is inconsistent in the literature and our findings, studies reported females at increased risk for burnout (Chen et al., 2025). Other international research confirms those results (Qu and Wang, 2015), while also emphasizing the role of age, children and the marital status as a risk factor for burnout among nurses (Cañadas-De La Fuente et al., 2015, 2018). Some studies have found that older age and more work experience are linked to higher risk of burnout and psychological stress (Xing et al., 2020). Other studies found that younger nurses were more likely to develop psychological disorders and older adults were less likely to experience burnout (Chen et al., 2025). Our findings indicated that the age of the participants, them being a parent or in a relationship did not have significant associations in our results. One potential explanation for our findings is that the relationship between age and burnout may be influenced by factors more closely related to nurses' day-to-day work experiences, and the overall psychosocial work environment. In our sample, these workplace factors may have had a stronger association with burnout than age itself. Furthermore, nurses of different ages may experience different types of work-related resources and challenges. Older nurses may have greater professional experience and coping resources, whereas younger nurses may have greater adaptability or different expectations regarding work and organizational support. These potentially opposing influences could attenuate an overall association between age and burnout. Other than that, it is possible that parenting can increase demands outside the workplace and perhaps even contribute to work-family conflict. However, it should be noted that parenthood can also be associated with social support, personal meaning, and coping resources. These findings suggest that age and parenting status alone may be insufficient to explain differences in burnout and that their potential effects may be better understood in the context of nurses' broader psychosocial work environment.

Our results confirmed that the nursing work field as examined in our study setting is still dominated by the image of the “classic” female, middle-aged nurse who completed three years of vocational training. This has also been confirmed by the German Federal Employment Agency reporting that more than four out of five nurses in Germany are female and two-third are under the age of fifty (Federal Employment Agency, 2025). For anyone who deviates from the norm in the ward team, it may be more difficult to fit into the given role structure and team context, thus indicating that change is still difficult to establish in the German nursing field. Our bivariate analysis confirmed that nurses born in another country than Germany experience less psychological safety in the team compared to their German-born colleagues. This can be due to communication issues and language barriers as well as difficulties adjusting to the new work environment. Studies have shown that migrant nurses continue to experience discrimination and reduced career opportunities in Germany and other high-income countries (Smith et al., 2022), thus possibly leading to our reported results.

We did see associations between our diversity factors and burnout in the bivariate analyses, but not in the multivariate analyses. Ultimately, this indicates that working conditions overshadowed the effect of personal diversity factors in our study. Even though it was not evident in our results, other studies reported that sufficient job resources such as feedback, autonomy and opportunities for growth and development could also be factors that prevent nurses from burning out (Kohnen et al., 2023).

Our results have shown that the burnout of the nurses is significantly associated with the personnel equipment and individual team structures on the ward. In practice, this suggests a focus on team development and optimizing the organizational framework conditions on the ward.

Interestingly, psychological safety within the immediate care team appears to be associated with personal burnout rather than interprofessional teamwork with the medical profession. The nursing team appears to be the relevant reference point, interfering with personal well-being. This also indicates where to address potential conflicts and how to improve management in practice.

Newer studies emphasize the interactions between psychological and organizational factors which significantly influence nurse burnout, creating a complex environment where both individual and workplace conditions contribute to burnout symptoms (Getie et al., 2025; Aljabri et al., 2022). This aligns with our findings of burnout being associated with different personal and organizational factors. More research will have to focus on this interaction to foster the ongoing diversification of the German nursing field. Focusing on recent nurse mental health struggles combined with the diverse nursing landscape and with individual post-pandemic work experiences, health organizations will need to regularly monitor the mental health status of its personnel. Regular team meetings, intercultural training and adequate staffing will enhance teamwork and ultimately the quality of patient care (Reiff et al., 2020).

### 8.2 Strengths and limitations

Despite the implementation of an innovative approach in the study's design, the study is not without its limitations. The present study was conducted in Berlin, Germany and at two different healthcare organizations. Given its cross-sectional design, it was not possible to undertake a longitudinal evaluation, and thus examination of effects on burnout. The study's observational and cross-sectional nature limited on a geographical area precluded the formulation of general statements from findings, as it was only possible to observe correlations and associations among the variables. Another limitation is the use of self-reported measures, which may be subject to response biases, like recall bias or social desirability bias. Although we used validated and reliable scales to collect this information, the lack of objective confirmation for all data points should be considered when interpreting the findings. Lastly, as access to individual email addresses of the nursing staff was not provided, multiple entries from the same participant are theoretically possible. Nevertheless, duplicate participation is highly unlikely as the survey was uncompensated, not credited as working hours, and required a substantial time investment. This could also explain the relatively low response rate that might indicate selection bias. However, despite these limitations, our study provided important key findings for the German nursing workforce with being the first to investigate the associations of intra- and interprofessional teamwork with burnout among diverse nursing teams and leading the way for more diversity sensitive research in this field. Future research should focus on longitudinal studies to assess the long-term impacts of intra- and interprofessional collaboration on burnout among nursing staff, providing deeper insights into how sustained collaborative practices influence mental health and retention rates over time. These studies should also examine the necessity for targeted staff development and organizational concepts to capitalize on the potential of heterogeneous teams and address the problem-oriented discourse.

## 9 Conclusions

Immigration and a (partial) shift of nursing toward a university degree are increasing the diversity of care teams in Germany and influencing intra- and interprofessional collaboration in day-to-day work. This study explores the simultaneous influence of diversity dimensions of nurses, their working conditions and social factors on burnout. Our analysis has shown that improved psychological safety and optimized staffing levels as well as social support in the form of private, close relationships improve burnout symptoms.

The interactions between professional and private stress need to be investigated in further longitudinal research.

We suggest the implementation of regular team meetings and collaborative platforms that could enhance communication among nursing staff, allowing for the sharing of experiences and strategies that address stressors, ultimately reducing feelings of isolation and burnout.

Establishing peer support initiatives within nursing teams might promote a culture of mutual assistance and understanding, which could significantly alleviate burnout by providing emotional support and practical solutions to common challenges faced in the workplace.

## Declaration of generative AI and AI-assisted technologies in the manuscript preparation process

During the preparation of this work the authors used DeepL Write in order to improve language and readability. After using this tool, the authors reviewed and edited the content as needed and take full responsibility for the content of the published article.

## Funding sources

This study was funded by the Federal Joint Committee of Germany (G-BA) (01VSF20001). The funders had no role in the study design, analyses, interpretation of data, writing the manuscript, approval, or decision to publish the results. No author was paid to write this article by any agency.

## CRediT authorship contribution statement

**Anna Maria Khouri:** Writing – review & editing, Writing – original draft, Visualization, Methodology, Investigation, Formal analysis, Data curation. **Jan C. Zoellick:** Writing – review & editing, Writing – original draft, Formal analysis. **Lisa Peppler:** Writing – review & editing, Project administration, Funding acquisition, Conceptualization. **Liane Schenk:** Writing – review & editing, Supervision, Funding acquisition, Conceptualization. **Anna Schneider:** Writing – review & editing, Writing – original draft, Methodology, Formal analysis, Data curation, Conceptualization.

## Declaration of competing interest

All authors declare that they have no conflict of interest to report.

## Data Availability

Due to ethical and legal restrictions defined in the approved ethics application, the datasets generated and analyzed during this study cannot be shared with third parties. Data sharing is therefore not permitted.
